# Detection of Vulnerable Atherosclerotic Plaque and Prediction of Thrombosis Events in a Rabbit Model Using ^18^F-FDG -PET/CT

**DOI:** 10.1371/journal.pone.0061140

**Published:** 2013-04-17

**Authors:** Quan-ming Zhao, Xin Zhao, Ting-ting Feng, Ming-duo Zhang, Xu-cui Zhuang, Xue-cheng Zhao, Li-qin Li, De-peng Li, Yu Liu

**Affiliations:** 1 Department of Cardiology, Beijing Anzhen Hospital, Capital Medical University, Beijing, China; 2 Center for PET/CT, General Hospital of Second Artillery of PLA, Beijing, China; 3 Institute of Basic Medicine, Capital Medical University, Beijing, China; University Medical Center Utrecht, The Netherlands

## Abstract

**Background:**

Detection of vulnerable plaques could be clinically significant in the prevention of cardiovascular events. We aimed to compare Fluorine-18 fluorodeoxyglucose (^18^F-FDG) uptake in vulnerable and stable plaques, and investigate the feasibility of predicting thrombosis events using Positron Emission Tomography/Computed Tomography (PET/CT) angiography.

**Methods:**

Atherosclerosis was induced in 23 male New Zealand white rabbits. The rabbits underwent pharmacological triggering to induce thrombosis. A pre-triggered PET/CTA scan and a post-triggered PET/CTA scan were respectively performed. ^18^F-FDG uptake by the aorta was expressed as maximal standardized uptake value (SUV_max_) and mean SUV (SUV_mean_). SUVs were measured on serial 7.5 mm arterial segments.

**Results:**

Thrombosis was identified in 15 of 23 rabbits. The pre-triggered SUV_mean_ and SUV_max_ were 0.768±0.111 and 0.804±0.120, respectively, in the arterial segments with stable plaque, and 1.097±0.189 and 1.229±0.290, respectively, in the arterial segments with vulnerable plaque (*P*<0.001, respectively). The post-triggered SUV_mean_ and SUV_max_ were 0.849±0.167 and 0.906±0.191, respectively in the arterial segments without thrombosis, and 1.152±0.258 and 1.294±0.313, respectively in the arterial segments with thrombosis (*P*<0.001, respectively). The values of SUV_mean_ in the pre-triggered arterial segments were used to plot a receiver operating characteristic curve (ROC) for predicting thrombosis events. Area under the curve (AUC) was 0.898. Maximal sensitivity and specificity (75.4% and 88.5%, respectively) were obtained when SUV_mean_ was 0.882.

**Conclusions:**

Vulnerable and stable plaques can be distinguished by quantitative analysis of ^18^F-FDG uptake in the arterial segments in this rabbit model. PET/CT may be used for predicting thrombosis events and risk-stratification in patients with atherosclerotic disease.

## Introduction

Atherosclerosis is a progressive and inflammatory disease characterized by the accumulation of lipids and fibrous elements in the arteries. The atherosclerotic plaque rupture and subsequent thrombus formation can cause severe complication such as myocardial infarction, or stroke [1]. Therefore, the early detection of these prone to-rupture plaques (vulnerable plaques) is clinically relevant to risk stratification and prevention of severe and acute atherosclerotic complications. Morphologically, a typical vulnerable plaque is characterized by a large, lipid-rich athermanous core, a thin fibrous cap, and infiltration by inflammatory cells, such as macrophages [2]. Several imaging approaches have been developed to detect atherosclerotic plaques. Conventional X ray-based contrast angiography simply images the lumen of the vessel, but it usually fails to indicate the atherosclerotic lesions that do not protrude into the lumen and it provides very little information on the composition of the atherosclerotic plaque. High-resolution intravascular imaging modalities, including virtual histology intravascular ultrasound, optical coherence tomography, and intravascular magnetic resonance imaging (IV-MRI), permit direct imaging of the plaques and vessel wall [3]. However, these invasive imaging methods require considerable time, expertise, and expense, and none is suitable for screening atherosclerosis and vulnerable plaques in asymptomatic patients.

Computed tomography angiography (CTA) [4] and high-resolution multicontrast MRI [5] allow the detection of coronary stenosis and atherosclerotic plaques, particularly when combined with appropriate intravenous contrast agents. However, vessel stenosis degree is not the determinant of plaque vulnerability, but the composition and inflammation state of the plaque play the key role [6]. Nuclear medical techniques can detect inflammation in atherosclerotic lesions and biopathological events. In this way, they constitute an excellent platform for the identification of vulnerable plaques on the basis of biologic characteristics [7]. For example, [18-F] fluorodeoxyglucose positron emission tomography/computed tomography (^I8^F-FDG PET/CT) has been used to detect inflammatory plaque and evaluate the therapeutic effect of anti-atherosclerotic drugs in animal experiments and clinical trials [8]–[12]. The aim of our study was to evaluate the role of PET/CT imaging in detection of vulnerable plaque and prediction of plaque rupture and thrombosis in a rabbit model.

## Methods

### Animal Model

Male New Zealand white rabbits (weight 2.8–3.5 kg, age 3 months, supplied by the Institute for Experimental Animals, Agricultural University of China) were used for the atherosclerotic model (n = 23). After fed with a 1.5% cholesterol diet for 2 weeks, the animals were subjected for a balloon injury at the abdominal aorta. The animals were then fed an intermittent high-cholesterol diet for 16 weeks (6 weeks of high-cholesterol diet +4 weeks of normal chow +6 weeks of high-cholesterol diet).

#### De-endothelialization of the aorta

Under sodium pentobarbital anesthesia, de-endothelialization of the aorta was performed by introducing a 4 F Fogarty arterial embolectomy catheter (Edwards Lifesciences, USA) via the right femoral artery. The catheter was advanced for 25 cm in a retrograde fashion to the ascending aorta, inflated with 0.5 ml water, and pulled back. This procedure was repeated 3 times. The femoral artery and skin were then closed in layers.

#### Pharmacological triggering

At the end of week 18, the 23 rabbits underwent pharmacological triggering to induce thrombosis within 24 hours. The rabbits were given Chinese Russell’s viper venom (RVV) 0.15 mg/kg (XinYuan Company, China) intraperitoneally, followed by histamine 0.02 mg/kg (Sigma, United States) via marginal ear vein injection in 30 min.

Procedures were performed according to the Capital Medical University Animal Care and Use Committee–approved protocol.

### *In vivo* PET/CT Imaging

A total of 34 PET/CTA scans were performed on the rabbits before and after the drug triggering (weight, 3.2±0.32 kg). Among the scans, 23 were obtained at week 18 after the induction of atherosclerosis (before drug triggering for thrombosis), and only 11 scans were obtained after drug triggering, because 12 rabbits died before the second PET/CT scan. ^18^F-FDG (1.0 mCi/kg) was injected intravenously to overnight fasted rabbits, and after 180 minutes PET/CT imaging was performed. Under general anesthesia with 3% sodium pentobarbital, the rabbits were held in a supine position on plastic boards with medical tape to minimize motion and then placed inside the PET/CT tube for scanning (GE Discovery ST, a fusion PET/CT scanner, clinically used equipment, spatial resolution 4–6 mm for PET). After a non-contrast CT examination, a 15 minutes PET 2-dimensional acquisition-per-bed-position scan was acquired for attenuation correction. An integrated 16-slice CT scan was performed on completion of PET imaging. The CT scan was performed (120 mA, 160 kV peak) using 1.2 mm collimation with continuous intravenous injection of 10 ml iopromide 350 (0.4 ml/s).

**Figure 1:**
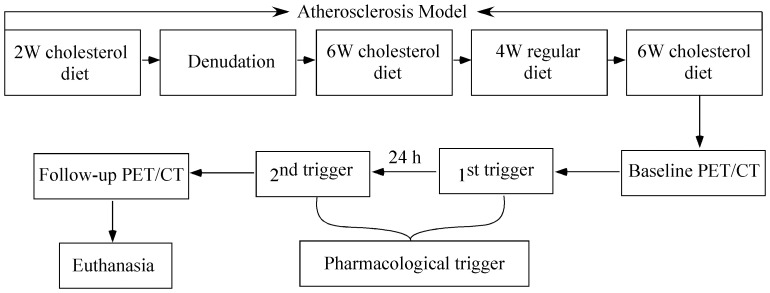
Schematic representation of pharmacological triggering and in vivo PET/CT examinations of the atherosclerotic rabbit model.

The time course of pharmacological triggering and *in vivo* PET/CT examinations of atherosclerotic rabbit model was illustrated in Figure 1.

### Gross Pathological Observation

Following the second PET/CT scan, the animals were sacrificed with an overdose of sodium pentobarbital (n = 11). The other 12 rabbits died after the drug triggering. The thoracic and abdominal aortae were subsequently removed and studied after perfusion fixation with PLP solution (75 mM L-(1)-lysine hydrochloride and 4% paraformaldehyde in 37.5 mM phosphate buffer, pH 7.4). The aortae were sliced into 7.5 mm segments by matching with the corresponding PET/CT images. The aortic segments were immediately postfixed in PLP solution.

### Image Analysis

Standardized uptake values (SUVs) were determined by a reader who was blinded to the histological and CT results, placing the region of interest over the stratum of the thoracic and abdominal aortae of each rabbit. SUVs were calculated on the workstation by averaging the two stratums of the selected segment with adjustment for subject weight, injected dose, and ^18^F-FDG decay. The specimen number was calculated in two ways: 7.5 mm aortic segments in each group and the numbers of the rabbits in each group.

### Histology

At necropsy, the aortae were dissected free and removed. The aortae and iliofemoral arteries were opened for inspection using a longitudinal incision. Arterial tissue sections (4 µm) were obtained from the aortic segments of 23 rabbits, yielding a total of 455 segments. The aortic segments were fixed in 10% buffered formaldehyde. Tissue sections were cut, embedded in paraffin on edge, and mounted on glass slides. Sections were stained with hematoxylin and eosin (H&E), and monoclonal antibodies for rabbit smooth muscle cell (SMC) (Thermo Corporation, USA), and rabbit macrophage CD-14 (Wuhan Boster Biological Technology Co. Ltd.). Tissue sections were examined with a light microscope (OLYMPUS BX41, Japan) and processed using CMIS of the pathology image analysis system (MOTIC). The thickness of fibrous cap and lipid core of each plaque were measured in the H&E-stained sections. Macrophage density was characterized for each CD-14 stained section as the number of macrophages per high power microscope field. SMC density was characterized for each SMC actin stained section as the number of the SMCs per high power microscope field. Macrophage density was used as an index of inflammation, and correlated with SUV in the aortic segments. The density of macrophages, density of SMCs, and ratio of the thickness of fibrous cap to the thickness of lipid core (cap-to-core ratio) [13] in each 7.5-mm segment were obtained by averaging the data at 3 positions (2, 4, and 6 mm).

The data for each 7.5-mm segment were used for comparison and correlation analysis with ^18^F-FDG uptake and SUV.

### Matching the PET/CT Slices with Histology

The distances from the renal branches and the iliac bifurcation were used as internal reference points to match the PET/CT slices with histology. During extraction, the aortas were marked with suture ligatures at regular distances above and below the left renal branch, over the total length imaged by PET/CT. After extraction, the ligatures were used to re-extend the aortas to their physiological length at the time when they were fixed with 10% formalin solution, cut in 7.5 mm segments, and matched with the PET/CT slices.

### Statistical Analysis

SPSS 13.0 for Windows was used for statistical analysis. Results were expressed as mean ± SD. The data sets were tested for normality using the Kolmogorov-Smirnov test with the Dallal-Wilkinson-Lilliefors correction, and were tested for equality of variances using the F test. Unpaired data were compared using the unpaired 2-sided t test. Correlation coefficients were assessed using Spearman rank correlation coefficients. A two-tailed value of *P*<0.05 was considered statistically significant. The receiver operating characteristic (ROC) curve was plotted to determine the SUV cutoff levels for predicting thrombosis. The level was set to obtain the maximum sensitivity plus specificity.

## Results

### PET/CT Imaging

**Figure 2:**
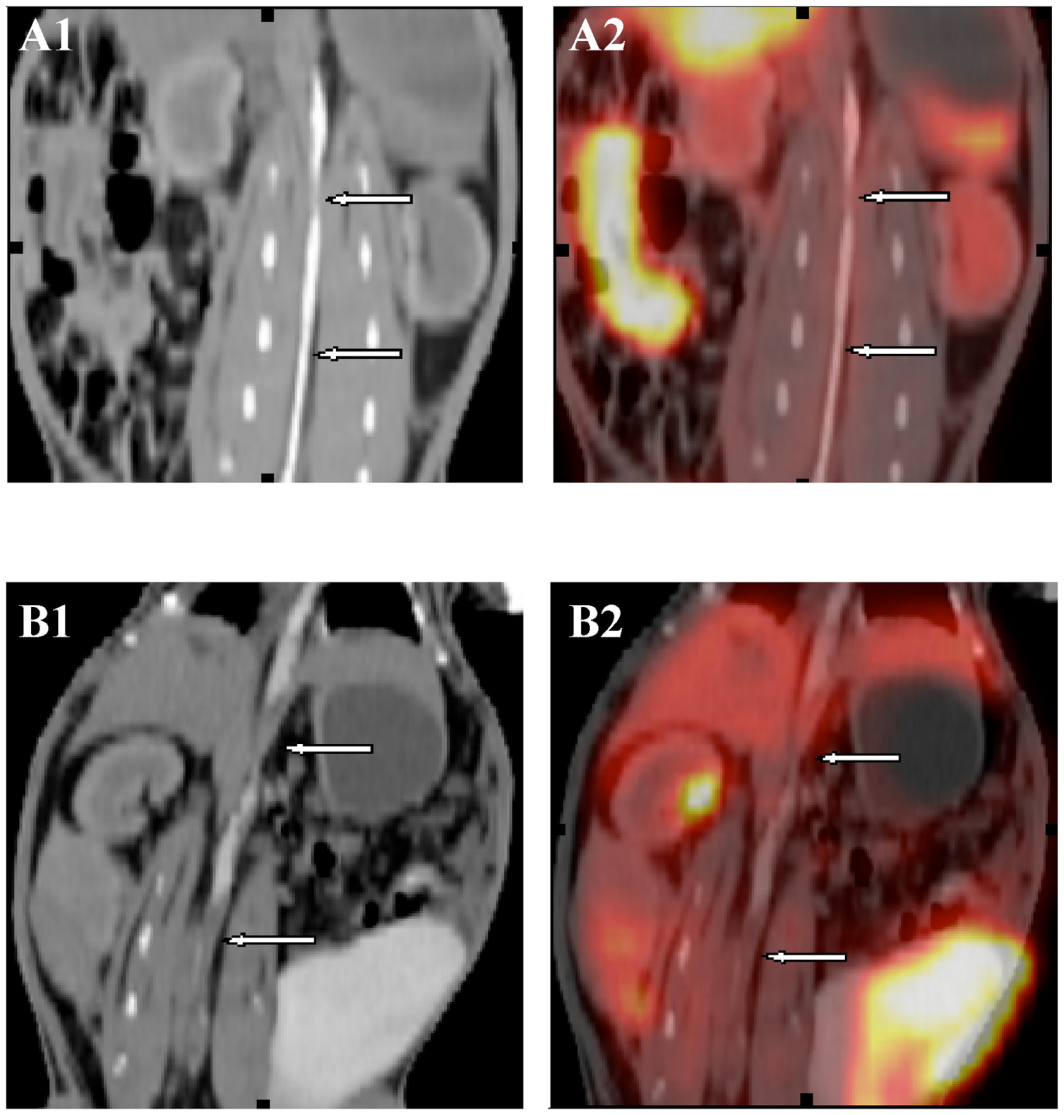
Representative aortic CT and PET/CT images of the atherosclerotic rabbits before and after pharmacological triggering. A1 and B1, Representative aortic images of CT angiogram before and after pharmacological triggering (respectively). A2 and B2, Representative aortic PET/CT fused images before and after pharmacological triggering (respectively). Luminal stenosis and irregularities on CT angiogram and evident FDG uptake on PET/CT images.

In order to evaluate the PET/CT imaging in detection of vulnerable plaque and prediction of thrombosis events we used a rabbit model for the experiments. The rabbits were first fed with high cholesterol diet for atherosclerosis model and then thrombosis was induced in these rabbits by injection of RVV (see Methods for details). The CT angiographic images and superimposed PET/CT images of the rabbit aortae were taken before and after pharmacological triggering and the results are shown in Figure 2 (A1 and A2, B1 and B2, respectively). In atherosclerotic aorta, the lumen stenosis and vessel wall irregularities are obvious on CT angiographic imaging (A1) and evident ^18^F-FDG uptake was observed on PET/CT images (A2). In the rabbits with thrombosis, luminal multi-defects can be seen on CT angiographic images (B1) and importantly much stronger ^18^F-FDG uptake was found on PET/CT images (B2). The results suggest that PET/CT is efficient to detect the ^18^F-FDG uptake at the atherosclerotic lesion and thrombosis.

### Gross Anatomical Analysis and Histo-pathological Analysis of the Rabbit Aortae

**Figure 3:**
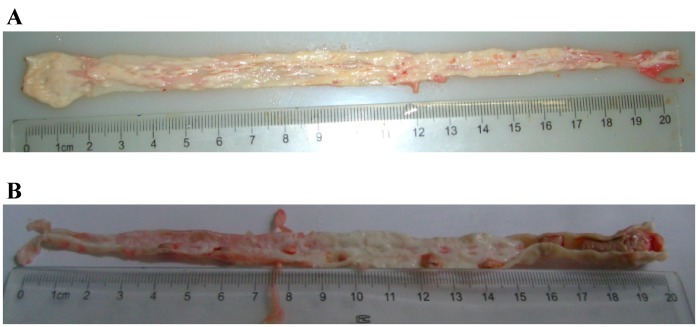
Representatives of aortae in the atherosclerotic rabbits after pharmaceutical triggering. A. Representative aorta with the thick intima and different plaques but no thrombosis formation after pharmaceutical triggering; B. Representative aorta with the thick intima, plaques and white thrombi after pharmaceutical triggering.

**Figure 4:**
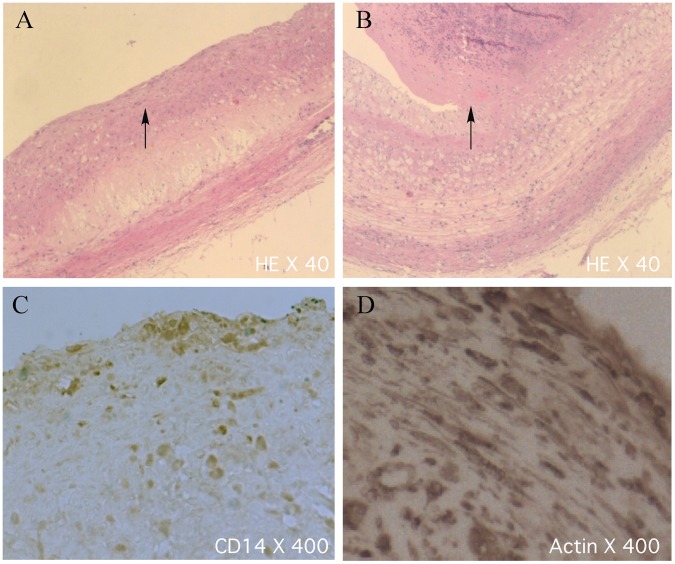
Histo-pathological images of the aortic atherosclerotic lesions. A and B. H&E staining sections of the stable plaque with thick fibrous cap and small lipid core, and the unstable plaque with fibrous cap disruption and thrombosis (respectively). C and D. Immunohistochemical staining sections with the antibodies against macrophage CD-14 and SMC actin (respectively). Macrophages were accumulated under fibrous cap.

To further investigate the atherosclerosis model and thrombosis triggering, we performed necropsy for the rabbits. The aortae were dissected longitudinally. It was found that atherosclerotic plaques were present in the entire aortic wall in the atherosclerotic rabbits (Fig. 3A). The plaques were more obvious below the renal arteries. The thrombi were identified in 15 of 23 rabbits (65%) after thrombosis triggering. The platelet-rich thrombi were found in the aortic segments where plaque rupture occurred (Fig. 3B). Stable plaques and vulnerable plaques could be differentiated on H&E staining (Fig. 4A and 4B). The stable plaques were capped by thick fibrous caps with small lipid cores while in the unstable plaque the fibrous caps were disrupted with thrombi. In a total of 455 segments, 65 were identified with thrombi. Staining the aortic atherosclerotic lesions with the antibodies against macrophages CD-14 and SMC actin reveals the presence of the large number of macrophage infiltration and the amplification of SMCs (Fig. 4C and 4D). All the data present here indicate that we established the rabbit atherosclerosis model and successfully induced the thrombosis by pharmacological triggering in those rabbits. Our histo-pathological analysis is able to differentiate the stable and vulnerable plaques.

### SUV Results and Analysis

**Figure 5:**
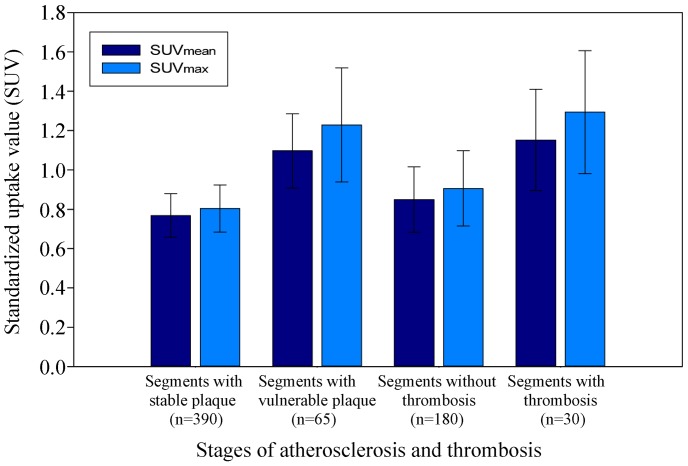
The SUVmean and SUVmax as measured by PET at different stages of atherosclerosis (per-segment analysis). Vertical lines represent SD.

The SUV data were obtained in a total of 455 aortic segments from 23 rabbits before thrombosis triggering (see Methods for details). The plaques from the aortic segments were classified into stable and vulnerable, based on the presence of luminal thrombosis seen on the post-triggered images and/or the corresponding histopathology. The plaques were defined as vulnerable plaques if there was luminal thrombosis. The results are shown in Table 1 and Figure 5. We first present the results from the rabbits before the thrombosis triggering. The SUV_mean_ and SUV_max_ were 0.768±0.111 and 0.804±0.120, respectively, in the arterial segments with stable plaques (n = 390), and 1.097±0.189 and 1.229±0.290, respectively, in the arterial segments with vulnerable plaques (n = 65). The results show that the values of SUV_mean_ and SUV_max_ from the vulnerable plaques are much higher than those from the stable plaques and the difference between two plaque groups was statistically significant (*P*<0.001) (Fig. 5).

**Table 1:** SUV_mean_ and SUV_max_ results (per-segment analysis).

	Pre-triggering		Post-triggering	
	Stable (n = 390)	Vulnerable (n = 65)	Non-thrombosis (n = 180)	Thrombosis (n = 30)
SUV_mean_	0.768±0.111	1.097±0.189¶	0.849±0.167	1.152±0.258†
SUV_max_	0.804±0.120	1.229±0.290¶	0.906±0.191	1.294±0.313†

¶ *P*<0.001 vs. Stable;

† *P*<0.001 vs. Non-thrombosis.

We also obtained the results for the ^18^F-FDG uptake in the aortic segments after pharmacological triggering. The SUV_mean_ and SUV_max_ were 0.849±0.167 and 0.906±0.191, respectively, in the arterial segments without thrombosis (non-thrombosis group, n = 180), and 1.152±0.258 and 1.294±0.313, respectively, in the arterial segments with thrombosis (thrombosis group, n = 30). Similar to the stable and vulnerable plaques, the values of the SUV_mean_ and SUV_max_ from the thrombosis group are higher than those from the non-thrombosis group and the differences were statistically significant (*P*<0.001) (Table 1 and Fig. 5). We also analyzed the effect of plaque rupture and thrombosis on^ 18^F-FDG uptake in aortic segments with vulnerable plaques. After drug triggering, SUV_mean_ and SUV_max_ in the arterial segments with thrombosis were each significantly higher than before drug triggering *(P*<0.001).

**Figure 6:**
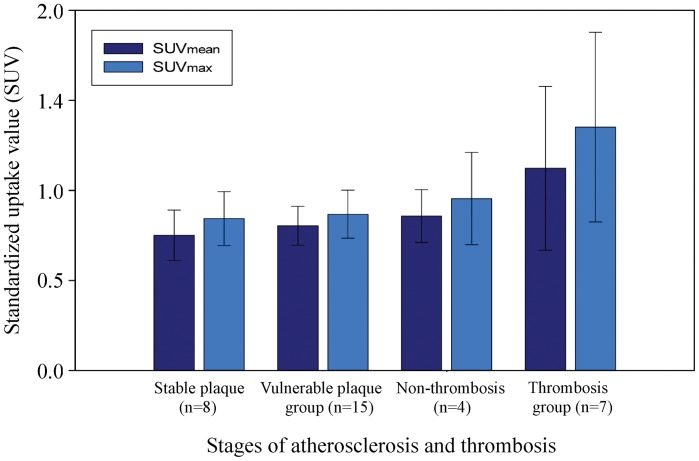
The average SUVmean and SUVmax as measured by PET at different stages of atherosclerosis (per-rabbit analysis). Vertical lines represent SD.

The SUV value per-rabbit was also analyzed. The average SUVmean and SUVmax were 0.751±0.140 and 0.844±0.150, respectively, for “stable plaques” group (n = 8); 0.804±0.108 and 0.868±0.134, respectively for “vulnerable plaques” group (n = 15), before pharmacological triggering. The average SUVmean and SUVmax were 0.858±0.147 and 0.955±0.256, respectively, for non-thrombosis group (n = 4); 1.230±0.455 and 1.352±0.527, respectively, for thrombosis group (n = 7 ), after pharmacological triggering (Fig. 6).

**Figure 7:**
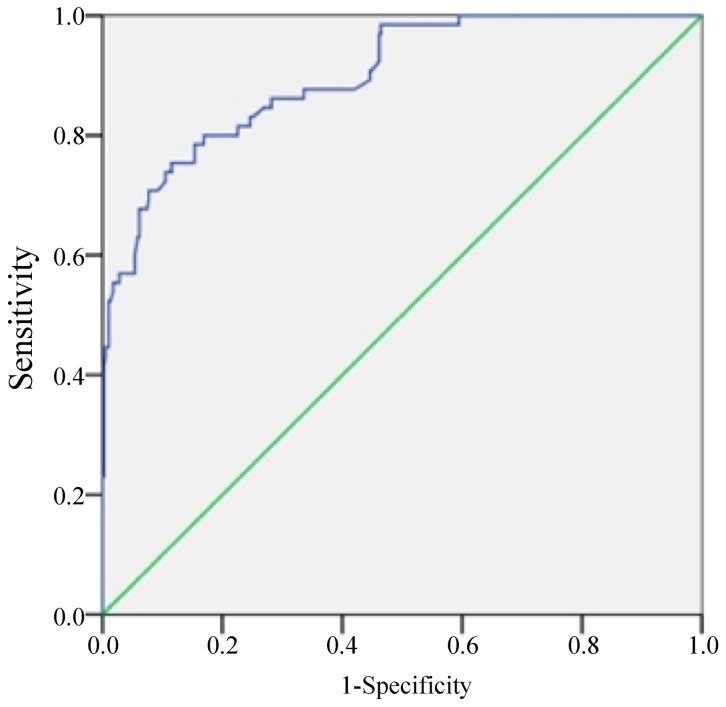
The ROC curve for SUV_mean_ to predict plaque rupture and thrombosis. Area under the curve (AUC) was 0.898 (*P* = 0.000). The cutoff value was 0.882.

To evaluate the SUV_mean_ cutoff values for predicting thrombosis events, we used the SUV_mean_ values in the arterial segments before drug triggering to plot a ROC curve (Fig. 7). Area under the curve (AUC) was 0.898 (95% *CI = *0.857–0.939) (*P* = 0.000), indicating that SUV_mean_ predicted thrombosis events. When SUV_mean_ was 0.882, sensitivity plus specificity was maximal. The sensitivity and specificity were 75.4% and 88.5%, respectively. If SUV_mean_ was ≥1.000, the predicting sensitivity and specificity were 53.5% and 98.5%, respectively.

### Correlation between ^18^F-FDG Uptake and Histological Findings

The relationship between FDG uptake in the arterial segments after drug triggering and the patho-histological parameters was analyzed. The cap-to-core ratio of the plaque from the morphological analysis was negatively correlated with SUV_mean_ (*r* = −0.276, *P*<0.001) and SUV_max_ (*r* = −0.274, *P*<0.001). SUV_mean_ was positively correlated with macrophage number (*r* = 0.316, *P*<0.001), but not correlated with SMC (*P* = 0.841). Similarly, SUV_max_ was positively correlated with macrophage number (*r* = 0.343, *P*<0.001), but not with SMC (*P* = 0.750).

## Discussion

An important mechanism responsible for the sudden and unpredictable onset of acute thrombosis is plaque rupture. The risk of rupture depends more on the plaque’s composition than its size [1]. Inflammation in plaque plays a critical role in determining plaque vulnerability [6].

### Atherosclerotic Animal Model with Vulnerable Plaques and Thrombosis

In an effort to identify suitable tools for vulnerable plaque detection, animal models of the atherosclerotic plaques are needed. The Constantinides New Zealand white (CNZW) rabbit model is widely used as an atherosclerotic animal model in experimental studies [14]. In this study in order to increase the rate of atherosclerosis without compromising the death rate, we chose an intermittent high cholesterol diet and de-endothelialization of the aorta. As reported by Abela GS et al [14], triggering of plaque disruption and thrombosis was accomplished by intraperitoneal injection of RVV, a procoagulant and endothelial toxin, followed by the intravenous injection of histamine, a vasopressor in rabbits. The aortae of the rabbits were found to have disrupted atherosclerotic plaques with overlying platelet-rich thrombi. Thrombosis was closely associated with the degree of atherosclerosis, and rarely occurred in normal blood vessels. Gross anatomical and histo-pathological analysis in our study demonstrated stable and vulnerable plaques in the specimens. Most types of atherosclerotic lesions reported by Phinikaridou et al were found in our model. These plaques were very similar to human vulnerable plaques. Thrombosis only occurred in vulnerable plaques (60% in ruptured plaque, and 40% in eroded plaques). Therefore, this modified model was considered ideal for atherosclerosis and thrombosis studies [15].

### FDG Uptake in Plaques can Distinguish Vulnerable and Stable Plaques

Among a number of non-invasive imaging modalities, multislice computed tomography (MSCT) is considered the most reliable means for detection of the size of coronary plaques and blood vessel stenosis [4]. However, conventional imaging techniques may not accurately detect the composition of the plaques and thus are not able to evaluate the high-risk of vulnerable atherosclerosis. Advances in our understanding of cell biology of atherosclerosis have led to the search for novel imaging strategies that can provide information about plaque composition and help understand the biological properties of atherosclerotic plaques. A number of noninvasive techniques for imaging of molecular or cellular targets have been explored. Among them, ^18^F-FDG-PET has been used to identify atherosclerosis in human studies [16].

Radiologists frequently use ^18^F-FDG to differentiate between benign and malignant tumors, and follow up the patients with cancers [17]. While investigating the mechanisms of FDG accumulation in tumor tissue, Kubota et al. reported that, within the tumor, the uptake of deoxyglucose by macrophages was higher than that by tumor cells [18]. Because atherosclerotic lesions are rich in macrophages, Vallabhajosula et al first applied FDG-PET in imaging of experimental atherosclerotic lesions of hypercholesterolemic rabbits in 1996, and found that the amount of FDG uptake in the lesion correlated with the macrophage density in the lesion [19]. Since then, serial studies have been carried out to explore the possibility for detection of atherosclerotic lesions and plaque inflammation with FDG-PET and PET/CT, in animal models and clinical trials [8]–[12], [20].

Our study indicated that the ^18^F-FDG uptake in the vulnerable plaques was significantly higher than in the stable plaques before drug triggering. Similarly, the ^18^F-FDG uptake in the plaques with thrombosis was significantly higher than in the plaques without thrombosis after drug triggering. Furthermore, the ^18^F-FDG uptake in the plaques associated with thrombosis after drug triggering was significantly higher than in the same arterial segments (vulnerable plaques) before the drug triggering.

Dynamic evaluation of ^18^F-FDG uptake in the same arterial segments is an important part of the animal model. The sole study on dynamic evaluation of ^18^F-FDG uptake in the CNZW model was reported by Aziz et al [21]. In their study, the average SUVmax was obtained in different stages of atherosclerosis (control group, the middle-of-feeding group, the end-of-feeding group, and triggered group). The difference among these groups was statistically significant. But there was no significant difference between the triggered subgroups that developed thrombus and the group that did not.

One of the important new findings from our study is that SUV_mean_ (an index of ^18^F-FDG uptake) can be used to distinguish vulnerable plaques from stable plaques before drug triggering. SUV_mean_ in the arterial segments with thrombosis was significantly higher than that in the arterial segments without thrombosis after the drug triggering. We believe this is the first report that SUV is an important determinant parameter to identify vulnerable plaques that would rupture or, where thrombosis would occur after drug triggering. It is known that the arterial segments where plaque rupture or thrombosis occurred had a higher degree of inflammation, and higher uptake of ^18^F-FDG.

We think that the difference between the results from this study and the previous report [21] came from the different methods used. In both studies the SUVs were measured and analyzed on the basis of the arterial segments, not on the number of rabbits. The SUV_max_ reported by Aziz et al [21] were obtained from three segments, the thoracic and the upper and lower abdominal aortae of each rabbit. The SUV_max_ and SUV_mean_ in our study were reported from the segments taken every 7.5 mm along the aorta. Approximately 30 segments and the same number of SUVs were obtained from each rabbit. The careful measures used for matching PET/CT slices with aortic section histology allowed accurate correlation of the two. Aziz et al reported the segments which were much longer than those in our study. The longer segments could contain both vulnerable and stable plaques. Thus it was difficult to distinguish vulnerable from stable plaques using that method.

ROC curve was used to evaluate the possibility for predicting plaque rupture or thrombosis events with SUV_mean_. AUC was 0.898, indicating that SUV_mean_ was a good index for predicting plaque rupture or thrombosis. The SUV_mean_ with a maximum sensitivity plus specificity was 0.882. The sensitivity and specificity were 75.4% and 88.5%, respectively. If SUV_mean_ was ≥1.000, the plaque was predicted to rupture or thrombosis occurred after drug triggering with a false positive rate less than 1.5%.

The positive correlation between the ^18^F-FDG uptake in the arterial segments and macrophage number indicated that the increased ^18^F-FDG uptake reflected inflammation in the arterial plaques. We used the cap-to-core ratio as an index of plaque stability, or an inverse index of plaque vulnerability [13]. The negative correlation between the ^18^F-FDG uptake and the cap-to-core ratio in our study demonstrated that the ^18^F-FDG uptake in the plaque was an index of plaque vulnerability.

However, certain factors must be taken into consideration when this method is applied in clinical settings. For example, the high macrophage content present in the plaques in this model could make detection of plaque inflammation easier with PET scanning. Also, in this case, plaque disruption or thrombosis were pharmacologically induced rather than natural. The threshold values of SUV indicate that predicting plaque rupture or thrombosis may be different under these experimental conditions and natural conditions. An additional difference is that the arteries imaged in this paper were aortae rather than coronary arteries. However, CT combined with PET imaging may be able to provide a superior level of details regarding the anatomy, size, and biological composition of plaques after problems with motion artifacts and image resolution have been resolved.

### Conclusions

Advances in our understanding of the biology of atherosclerosis have highlighted a clear need for noninvasive imaging techniques that can provide information about plaque composition, which is the major determinant of plaque stability. Our PET/CT studies in a rabbit atherosclerosis model suggest that ^18^F-FDG PET may provide invaluable information regarding the cellular, metabolic, and molecular composition of these plaques and thus distinguish vulnerable plaques from stable ones.
